# Factors of wheat frost hardiness – ice recrystallization inhibitor proteins

**DOI:** 10.18699/vjgb-26-43

**Published:** 2026-05

**Authors:** N.Е. Korotaeva, А.V. Fedyaeva, К.К. Musinov, А.S. Surnachev, G.B. Borovskii

**Affiliations:** Siberian Institute of Plant Physiology and Biochemistry of the Siberian Branch of the Russian Academy of Sciences, Irkutsk, Russia Institute of Cytology and Genetics of the Siberian Branch of the Russian Academy of Sciences, Novosibirsk, Russia; Institute of Cytology and Genetics of the Siberian Branch of the Russian Academy of Sciences, Novosibirsk, Russia; Institute of Cytology and Genetics of the Siberian Branch of the Russian Academy of Sciences, Novosibirsk, Russia Siberian Research Institute of Plant Production and Breeding – Branch of the Institute of Cytology and Genetics of the Siberian Branch of the Russian Academy of Sciences, Krasnoobsk, Novosibirsk region, Russia; Institute of Cytology and Genetics of the Siberian Branch of the Russian Academy of Sciences, Novosibirsk, Russia Siberian Research Institute of Plant Production and Breeding – Branch of the Institute of Cytology and Genetics of the Siberian Branch of the Russian Academy of Sciences, Krasnoobsk, Novosibirsk region, Russia; Siberian Institute of Plant Physiology and Biochemistry of the Siberian Branch of the Russian Academy of Sciences, Irkutsk, Russia

**Keywords:** ice recrystallization inhibition proteins, Triticum aestivum L., winter hardiness, regulation of gene activity, белки-ингибиторы рекристаллизации льда, Triticum aestivum L., зимостойкость, регуляция активности генов

## Abstract

For winter wheat, winter hardiness is one of the complex traits that determine the successful cultivation of this crop, and the responsible genes are recognized as highly significant for breeding work. The accumulation of proteins that prevent ice recrystallization (ice recrystallization inhibition proteins, IRIP) correlates with the survival of winter wheat, which indicates the importance of taking this trait into account when obtaining more frost-resistant varieties. The importance of IRIPs is determined by their ability to integrate into growing ice crystals, which limits the formation of large ice conglomerates in the tissues of winter plants. Wheat IRIPs, which accumulate mainly in the apoplast of leaves and in the crowns during cold acclimation, are characterized by a typical duality of structural organization that determines both the manifestation of IRI activity and anti-pathogenic properties. The wheat IRIP molecule contains at the C-terminus a conserved NxVx(x)G fragment that repeats several times, forming a β-helix responsible for binding to the ice surface; at the N-terminus, there is an LRR sequence typical of pathogen-activated kinases, as well as a guiding signal peptide. The wheat genome contains up to eleven IRI genes. The TaIRI gene promoter contains typical basic cis-activating elements and some elements that respond to abiotic stress and hormones. Isoforms of proteins responsible for protecting against pathogens (pathogenesis related proteins, PRP), which accumulate in winter wheat during cold acclimation, also have IRI activity. The expression of the IRIP and PRP genes positively correlates with the cold resistance of winter wheat plants. According to modern data, the regulation of the IRIP genes and cold-activated PRP genes is ABA-independent, but depends on the presence of jasmonic acid and on some proteomic transcription factors. The review provides examples of the practical use of isolated winter wheat IRIPs. The issue of the factors regulating the activity of the IRIP genes and cold-activated PRPs is the least developed to date. The association of these proteins with the winter hardiness of wheat indicates the prospects for their further study.

## Abstract (ru)

Для озимой пшеницы зимостойкость – один из комплексных признаков, определяющих успешное возделывание этой культуры, а отвечающие за нее гены признаны высоко значимыми для селекционных работ. Накопление белков, препятствующих рекристаллизации льда (ice recrystallization inhibition proteins, IRIP), коррелирует с выживаемостью озимой пшеницы, что указывает на важность учета этого признака при получении более морозоустойчивых сортов. Значение IRIP для выживаемости определяется их способностью встраиваться в растущие кристаллы льда, что ограничивает формирование крупных ледяных конгломератов в тканях озимых растений. IRIP пшеницы, накапливающиеся преимущественно в апопласте листьев и узлов кущения в период холодовой акклимации, характеризуются типичной двойственной структурной организацией, определяющей как проявление IRI-активности, так и антипатогенные свойства. Молекула IRIP пшеницы содержит на С-конце консервативный несколько раз повторяющийся фрагмент NxVx(x)G, формирующий ответственную за связывание с поверхностью льда β-спираль; на N-конце расположены типичная для активируемых патогенами киназ LRR-последовательность, а также направляющий сигнальный пептид. Геном пшеницы содержит до 11 IRI-генов. Промотор генов TaIRI содержит типичные основные цисактивирующие элементы и некоторые элементы, реагирующие на абиотический стресс и гормоны. Изоформы ответственных за защиту от патогенов белков (pathogenesis related proteins, PRP), накапливающихся у озимой пшеницы в период холодовой акклимации, также обладают IRI-активностью. Экспрессия генов IRIP и PRP положительно коррелирует с холодоустойчивостью растений озимой пшеницы. Регуляция генов IRIP и генов PRP, активируемых холодом, согласно современным данным, АБК-независимая, но зависит от присутствия жасмоновой кислоты и от некоторых протеомных факторов транскрипции. В обзоре приведены примеры практического использования изолированных IRIP озимой пшеницы. Вопрос о факторах регуляции активности генов IRIP и активируемых холодом PRP является наименее разработанным на сегодняшний день. Связь этих белков с зимостойкостью пшеницы указывает на перспективность их дальнейшего изучения.

## Introduction

Winter wheat is an economically valuable cereal crop that is of special interest for plant breeders. This crop is cultivated worldwide in regions with significantly different combinations of climatic conditions, which requires a highly diverse selection of high-yielding, regionally adapted varieties. The availability of a large set of studied traits and their associated genes significantly expands the potential for regional adaptation of wheat and for increasing its yield under specific regional conditions.

Active vegetative growth ensuring high yield of winter wheat depends on the overwintering success rate of winter cereal crops. Traits ensuring high survival during the winter-spring period are of special importance. Winter hardiness is a complex trait responsible for stability against several environmental stresses, including soil freezing (Ambroise et al., 2020).

Soil freezing, which leads to ice formation in the crowns of winter wheat, is one of the most significant damaging factors contributing to survival and yield reduction. The accumulation of antifreeze proteins (AFPs) protects the crowns and leaf parts of the plants from freezing and acts as an indicator of increased frost hardiness. The results of studies on AFPs in cereals were presented earlier in the review by J.G. Duman and M.G. Wisniewski (2014). The reviews available to us mostly focus either on cold tolerance or AFPs in plants in general. The information obtained so far on these proteins in wheat remains fragmented.

The goal of this paper is to provide a comprehensive overview of the currently available literature data on AFPs in soft wheat Triticum aestivum L. Here, a general characterization of these proteins will be presented, their molecular organization at the proteomic and genomic levels will be examined, and current knowledge on the regulation of their gene activity and practical applications of AFPs in T. aestivum will also be presented.

## General characterization of antifreeze proteins

AFPs were first described in Antarctic fish more than fifty years ago (DeVries, Wohlschlag, 1969). Since then, the body of knowledge about these proteins has significantly expanded, and methods for their practical application have been proposed and tested (Voets, 2017; Liu et al., 2021). Several terms are used in research papers to refer to these proteins, including ice-structuring proteins (ISP) (Kontogiorgos et al., 2007; Wang X. et al., 2024), ice-binding proteins (IBP) (Janech et al., 2006), thermal hysteresis proteins (THP) (Ewart et al., 1999), or ice recrystallization inhibitor proteins (IRIP) (Knight et al., 1995). Some plant AFPs are glycosylated proteins, commonly referred to as antifreeze glycoprotein proteins (AFGP) (Griffith et al., 1992). The term “IRI proteins” (IRIP) is currently accepted for wheat AFPs with annotated genes, as it largely reflects their functional activity (Wisniewski et al., 2020).

AFPs in plants were first described in bittersweet nightshade (Urrutia et al., 1992) and winter rye (Griffith et al., 1992). It was later found that plant AFPs had a specific distinctive feature compared to those of other living organisms. Plant AFPs are characterized by a weak ability to lower the freezing point of water below its melting point, a property known as thermal hysteresis. Only a few plant species exhibit thermal hysteresis up to +2 °C (Jarząbek et al., 2009; Bayer-Giraldi et al., 2011). In contrast, this property is strongly expressed in animal AFPs (Kristiansen et al., 1999, 2011). In wheat leaves, thermal hysteresis measured in February is about 0.2 °C (Duman, Olsen 1993).

Ice recrystallization occurs during one of the water freezing stages, when latent heat is slowly released as a result of ice formation (Puchkov, 2017). The ability of plant IRI proteins (IRIPs) to inhibit ice recrystallization lies in the binding of a protein molecule to the surface of a growing ice crystal and its incorporation into the crystal, which shapes the crystal as a hexagonal disk (at low IRIP concentrations) or a bipyramidal prism (at high IRIP concentrations) (Grifﬁth et al., 2005). Importantly, this prevents the formation of large ice conglomerates (Yang et al., 2024), which are likely to damage the living tissues.

The level of IRI activity depends on the concentration of IRIPs in the freezing solution (Regand, Goff, 2006). The ability of plant IRIPs to inhibit ice recrystallization was first described in rye (Griffith et al., 1992) and later in other cold-tolerant species (Antikainen, Griffith, 1997). This property turned out to only be characteristic of frost-hardy plants (Hon et al., 1995; Antikainen, Griffith, 1997).

As an overwintering cold-tolerant species, wheat contains IRI genes. The accumulation of IRIPs in wheat is especially relevant in late autumn and early winter, preceded by cold acclimation at low positive temperatures. At temperatures ranging from –6 to –8 °C, cold-acclimated wheat plants exhibit intercellular ice formation in the vascular transition zone of crowns, where large quantities of water migrate from the meristem zone. This water redistribution contributes to the better protection of the meristem zone from ice damage and ensures winter survival (Willick et al., 2019). IRIPs accumulate primarily in the vascular transition zone of crowns compared to the stem meristem zone (Willick et al., 2018).

It is worth noting that the minimum temperature at which meristematic cells survive in the overwintering wheat is close to the LT50 (Lethal Temperature 50 – the temperature at which 50 % of the test plant population dies), whereas IRIP accumulation correlates with the overall winter survival (Chun et al., 1998). In spring, IRI genes affect growth, heading, and the onset of flowering in wheat by stimulating the activity of genes responsible for vernalization (Cao et al., 2021).

Snow cover creates favorable conditions for the survival of pathogenic fungi (Gaudet, Laroche, 1997). As a result of tissue damage caused by growing ice conglomerates, there is a high risk of pathogenic infection in winter wheat plants after the loss of snow cover at the end of winter. In response to pathogen attack, plants synthesize PR proteins (PRPs), which are released into the apoplast upon infection to inhibit fungal enzymes or enzymatically degrade their cell walls (Jain, Khurana, 2018).

The LRR amino acid sequence, which is common among PRPs, is also contained in wheat IRIP molecules (Tremblay et al., 2005), where it is responsible for the PR activity (Juurakko et al., 2022). Some wheat PRPs, such as β-1,3-glucanases, chitinases, thaumatin-like proteins, and polygalacturonase-inhibition proteins, exhibit antifreeze activity (Hon et al., 1995; Kontogiorgos et al., 2007). In response to cold, wheat accumulates specific PRP isoforms acting as IRIPs, modifying ice growth during freezing, and ensuring pathogen resistance even before infection (Grifﬁth, Yaish, 2004).

Wheat IRIPs have been found in plant organs such as roots, stems, leaves, and crowns (Duman, Olsen, 1993; Willick et al., 2018, 2019; Jin et al., 2022; Vaitkevičiūte et al., 2024). The initial activation of TaIRI genes after cold exposure occurs in the leaves and then propagates to roots and stems (Jin et al., 2022), which is likely due to leaves being above-ground organs, and as such, the first to be affected by freezing. For example, the TaIRI1 transcript was detected in leaves, crowns, and roots, while the TaIRI2 transcript was only found in leaves (Tremblay et al., 2005).

A significant accumulation of IRIPs in the extracellular space is observed in winter cereals during cold acclimation (Antikainen, Grifﬁth, 1997). PRPs, the genes of which are regulated by cold acclimation/deacclimation, are likely to act as IRIPs and may be localized in the extracellular space, plasma membrane, nucleus, or cytoplasm (Vaitkevičiūte et al., 2024).

## Amino acid sequence of T. aestivum IRIPs

It was previously shown that the mature T. aestivum IRIPs TaIRI-1 and TaIRI-2 with molecular masses of 26.8 and 40.7 kDa respectively, share 40.4 % sequence identity (Tremblay et al., 2005). A conserved fragment located at the C-terminus and consisting of NxVxxG or NxVxG repeat motifs, where “x” represents a nonconserved residue, is recognized as the main conserved fragment of plant IRIPs, including those of wheat (Tremblay et al., 2005; Middleton et al., 2012; Jin et al., 2018). This conserved amino acid sequence, repeated several times at the C-terminal region, forms a β-helix structure. The two sides of this helix make it possible for IRIPs to bind to the surfaces of two forming ice crystals (Middleton et al., 2009).

Presumably, the number of repeats of this conserved sequence evolved through duplication events during the acquisition of cold tolerance in plants, and an increased number of repeats is likely associated with enhanced resistance to ice formation (Sandve et al., 2008). The number of repeats of the conserved C-terminal motif may vary. For example, TaIRI-1 and TaIRI-2, the two IRIPs of T. aestivum, exhibit five and six repeats, respectively (Tremblay et al., 2005). In a later study, the six identified T. aestivum IRIPs were found to contain 9 to 13 repeats (Jin et al., 2018).

The N-terminal region of IRIPs contains a leucinerich segment (Jin et al., 2018). The LRR amino acid sequence, which is common among both eukaryotic and prokaryotic organisms, is known to determine the properties of protein-protein and ligand-binding interactions. In plants, it is incorporated into protein molecules synthesized in response to stress caused by pathogenic organisms (McHale et al., 2006). The LRR domain of T. aestivum proteins TaIRI-1 and TaIRI-2 turned out to be homologous to receptor-like kinases (Tremblay et al., 2005) involved in signaling pathways triggered by the emergence of pathogen-associated elicitor molecules (He, Wu, 2016).

Another region of TaIRI molecules turned out to be homologous to the receptor kinase of the phytosulfokine peptide phytohormone (Tremblay et al., 2005) involved in differentiation of the plant immune response depending on the pathogen type (Sauter, 2015). T. aestivum proteins coded for by genes Tr001_B19 and Tr001_M19 showed high similarity to cold-regulated IRIP sequences and were annotated as protein kinases with LRR sequences (Monroy et al., 2007). Although IRIPs with kinase domains do not exhibit protein kinase activity (Tremblay et al., 2005), these findings indicate a similarity between IRIPs and PRPs. The N-terminal region of T. aestivum IRIPs also contains a signal peptide directing proteins to the extracellular space or to the membrane (Tremblay et al., 2005).

Eight wheat IRIPs were grouped based on the similarity of their amino acid sequences. According to phylogenetic analysis, the amino acids located at the C- or N-terminal regions determine the classification of some wheat IRIPs into group 2 (TaIRI-6, TaIRI-7, and TaIRI-8). Group 1 includes the proteins TaIRI-1, TaIRI-3, TaIRI-4, and TaIRI-5. TaIRI-2 protein having a distinct LRR domain was attributed to the third group (Jin et al., 2018).

## T. aestivum IRIP gene organization

The IRI gene family in cold-tolerant grasses of the Pooideae subfamily began to form and acquire specific features approximately 75 Mya, following the divergence of this subfamily into a separate taxon (Sandve et al., 2008). According to the prevailing evolutionary hypothesis with regard to LRR-IRI-like genes, the most recent common ancestor of IRI genes in Pooideae was the OsLRR-PSR-like gene identified in rice. This gene contained at its 5′ end an LRR sequence characteristic of the phytosulfokine receptor kinase. Notably, this sequence is also present in TaIRI genes (Houde et al., 2006). It is assumed that the IRI-coding fragment appeared in TaIRI genes as a result of an insertional event (Tremblay et al., 2005). The major expansion of the IRI gene group in Pooideae occurred around 36 Mya due to duplications of a specific IRI region within the genes (Sandve et al., 2008). In various studies, T. aestivum was found to possess two (Tremblay et al., 2005), six (Jin et al., 2018), or eleven IRI genes (Sandve et al., 2008) containing both LRR and IRI coding fragments. The sequence identity between TaIRI1 and TaIRI2 is 74.7 and 54.1 % at the 5′ and 3′ ends, respectively (Tremblay, 2005).

With few exceptions, the grouping of IRI genes in monocotyledons based on genomic sequence similarity generally corresponds to genus affiliation and phylogenetic relationships between the taxons. It is noteworthy that the organization of IRI genes in Triticum is closely related to that of Aegilops genus plants, which are the progenitors of allohexaploid wheat. Differences in characteristic regions of TaIRI (T. aestivum) gene sequences were found when compared against TdiIRI1 (T. dicoccoides), TmIRI1 (T. monococcum) and TuIRI1 (T. urartu), but not against TdIRI1 (T. durum) (Jin et al., 2018).

Eight TaIRI gene sequences are divided into four groups, with one gene each in groups III and IV (TaIRI6 and TaIRI2, respectively), four genes in group I (TaIRI1, TaIRI3, TaIRI4, and TaIRI5), and two genes in group II (TaIRI7 and TaIRI8) (Sandve et al., 2008; Jin et al., 2018).

The promoters of TaIRI genes contain typical core cis-acting elements as well as elements responsive to abiotic stress and hormones. These responsive elements include three elements in charge of responses to low temperature and drought stresses, one element specifically in charge of stress response at low temperatures, two elements involved in methyl jasmonate signaling response, four light-responsive elements, one abscisic acid-responsive element (ABRE), and several additional regulatory elements (ARE, CAT-box, O2-site and TCrich repeats) (Jin et al., 2022).

## Mapping of T. aestivum IRIP and cold-activated PRP genes

To date, a substantial amount of information has been obtained regarding the chromosomal localization of traits associated with wheat frost hardiness. Relevant traits for frost hardiness are located in QTL (Quantitative Trait Loci) regions on chromosomes 1D, 2A, 2B, 6D, and 7B (Båga et al., 2007); 5B and 5D (Chun et al., 1998); 5A, 2D, 2A, and 4B (Case et al., 2014); 5A and 4B (Kruse et al., 2017); as well as in chromosomes from group 5, 2B, and 4B (Sutka, 1994, 2001). The Fr1 and Fr2 genes controlling frost hardiness have been mapped on chromosomes 5A and 5D, respectively (Sutka et al., 1997). Substitutions of chromosomes from the Cheyenne variety for chromosomes 4A, 6A, 3B, 5D, and 3D of the frost-sensitive Chinese Spring variety resulted in increased protein accumulation in the apoplast, while the substitutions of chromosomes 1A, 5A, 4B, 5B, 6B, 1D, and 5D resulted in increased antifreeze activity.

The key regulatory genes responsible for increasing both the antifreeze activity and protein accumulation in the leaf apoplast were observed on chromosomes 5B and 5D (Chun et al., 1998). Two TaIRI genes were mapped on the same chromosomes obtained from the leaves of the cold-acclimated Zhoumai variety of T. aestivum (Zheng et al., 2020). The Tr015_M14 and Tr017_M15 genes coding for IRIPs in the Chinese Spring variety, were located in the 4A locus (Monroy et al., 2007). Notably, the analysis was conducted using plant parts that included both the crown and leaves, despite the fact that the highest IRIP content in wheat plants exposed to freezing is found in the vascular transition zone of crowns, while the meristematic zone and leaves exhibit their own specific responses to freezing (Willick et al., 2018).

Since IRIPs contain a fragment related to PRPs, it is reasonable to search for loci that simultaneously control responses to cold and pathogen attack. QTL regions in T. aestivum associated with resistance to both stress factors have been identified on chromosome 5A and are closely linked to the Fr2 locus. Separate QTLs associated with freezing hardiness were found on chromosomes 5A and 4B, while a QTL associated with snow mold resistance was identified on chromosome 6B (Kruse et al., 2017). The accumulation of β-1,3-glucanases, chitinases, and thaumatin-like proteins under freezing conditions was reduced in plants with chromosome substitutions 2A, 3A, 6B, and 7A. Substitution of chromosome 7A led to a significant prolongation of the freezing period followed by PRP gene activation (Chun et al., 1998).

It can be seen that the composition of QTL regions responsible for frost hardiness and antifreeze activity in wheat varies among varieties. In addition, the specific plant part used for mapping plays a significant role. Therefore, when genome organization of wheat plants is analyzed in studies of proteins with IRI activity, it is essential to take these factors into account.

## Effect of low temperatures on the activity of T. aestivum IRIP genes

To date, substantial evidence has been accumulated showing that the expression of T. aestivum AFPs is induced by cold exposure (see the Table) and is associated with cold and frost hardiness of the variety

The expression of three TaIRI genes (TaIRI16, TaIRI17, and TaIRI18) in stem, leaf, and root tissues of young T. aestivum plants was induced by cold (+4 °C, from 2 to 72 hours), and TaIRI16 showed the most significant induction among them (Jin et al., 2018, 2022). The TaIRI1 transcript in soft wheat begins to accumulate after low temperature exposure (+4 °C) and reaches its maximum level after 36 days of acclimation. Further exposure to low temperature does not lead to a higher transcript level. The TaIRI2 transcript also accumulates immediately after the onset of cold hardening, but it reaches its peak earlier in the acclimation period. After deacclimation, transcript levels return to those of non-acclimated plants (Tremblay et al., 2005).

Cold hardening (1–6 days) of seedlings from the Jagger and Alabaskaya varieties showed increased transcript levels of the Tr001_B19 (GB ID CK197231) and Tr001_M19 (GB ID CK201227) genes exhibiting high sequence similarity with cold-regulated IRIPs (Monroy et al., 2007). The activity of TaIRI genes related to genes coding for Lolium perenne IRIPs more than doubled after moving cold-hardened T. aestivum plants to subzero temperatures (Herman et al., 2006). The activity of the AFP I precursor gene (represent sequence ID CK197682) was more than eight times as high within one day of freezing young wheat plants at –5 °C and reached even higher levels by the third day of freezing (Kang et al., 2013).

Levels of TaIRI-1 and TaIRI-2 transcripts increase significantly in response to both gradual and abrupt temperature drops. Their induction occurs later than that of PRP-encoding genes. The highest accumulation levels are observed in leaves, especially when it comes to the TaIRI-2 transcript (Winfield et al., 2010).

Much of the evidence for the role of IRIPs in cold and frost hardiness has been obtained using wheats varying in their cold tolerance. Enhanced expression of TaIRI-1 and TaIRI-2 in response to low temperatures was observed in winter wheat cultivars compared to spring ones (Winfield et al., 2010). The more cold-tolerant cultivar Mironovskaya 808 has demonstrated higher TaIRI gene activity under all tested cooling regimes (Jin et al., 2022). After the first six hours of cold acclimation, transcript levels of Tr015_M14 and Tr017_M15 coding for AFPs were lower in the winter cultivar CDC Clair (LT50 –17 °C) than in the spring cultivar Quantum (LT50 –8 °C). However, the gene activity in the winter cultivar exceeded that in the spring cultivar after the first day of hardening, with almost three times the difference in Tr017_M15. Expression of the Tr015_J17 gene, annotated as IRIP-encoding, was two times as high in winter wheat seedlings after one day of acclimation at +4 °C compared to the spring cultivar, and this difference increased with longer acclimation up to day 14. Transcript levels of Tr001_B19 in the winter cultivar CDC Clair exceeded those in the spring cultivar Quantum at all stages of acclimation (from 6 hours to 14 days) (Monroy et al., 2007).

Additional evidence for the role of IRIPs in T. aestivum cold tolerance was obtained through gene transfer experiments. Expression of the TaIRI4 and TaIRI6 genes from the frost-resistant cultivar Mironovskaya 808 in transgenic tobacco under freezing conditions led to reduced membrane permeability (with the transformation of TaIRI4) and decreased lipid peroxidation activity (with transformations in both TaIRI4 and TaIRI6) (Jin et al., 2018).

These studies provide convincing evidence that the expression of T. aestivum IRIP genes is activated by both subzero and low positive temperatures and contributes to the development of frost hardiness in this species

## Regulation of T. aestivum PRP gene activity by low temperatures

A number of PRP-encoding wheat genes are activated by low temperatures (see the Table). It is assumed that such genes code for PRP isoforms with IRI activity. M. Houde et al. (2006) obtained high-quality ESTs (Expressed Sequence Tags) from wheat cDNA libraries associated with cold stress. A set of 75,488 unique sequences (31,580 contigs and 43,908 singletons/singlets) enriched with stress-regulated genes was obtained. Among them were ESTs containing contigs coding for chitinases, β-1,3-glucanase, and thaumatin-like proteins. Fifteen of these sequences were annotated as precursors or actual chitinases, six of which being cold-inducible. The transcript level of the TaGLB2b gene coding for glucanase significantly increases only in response to gradual temperature decrease, while no increase is observed under sudden cold shock. Its induction occurs earlier than that of TaIRI genes, i. e. between the third and the fifth weeks of cooling (Winfield et al., 2010).

The activity of the JC5845 gene coding for chitinase more than doubles after moving cold-hardened T. aestivum plants to subzero temperatures (Herman et al., 2006). The gene coding for chitinase 3 (represent sequence ID AB029936.1) was among those, the activity of which was more than eight times as high in response to freezing young wheat plants at –5 °C. Its expression level rose significantly depending on the duration of exposure, with nearly a 20 times difference between the measurements taken on days 1 and 3 of freezing (Kang et al., 2013).

Differences in PRP gene expression levels in response to cooling were observed between wheats varying in PR and frost hardiness. When comparing the genomes of spring and winter wheat cultivars under cold acclimation (+4 °C, from 6 hours to 14 days), no significant differences were found in the activity of genes coding for thaumatin or chitinase. However, the increase in transcript levels of the Tr017_C17 gene coding for chitinase II precursor in response to hardening was higher in the winter cultivar (Monroy et al., 2007).

A number of chitinase and β-1,3-glucanase genes are regulated by autumn cold acclimation: their expression begins in late autumn, peaks in mid-winter, then decreases, and reaches maximum levels again in spring. Under cold acclimation, β-1,3-glucanase gene transcripts are weakly expressed during short-term exposure, but the expression increases significantly with prolonged exposure and remains elevated after returning to control conditions. The chitinase-encoding gene begins active expression immediately after the onset of cooling, and its expression decreases after returning to control conditions. Differences in expression levels of these genes between cultivars resistant and less resistant to snow mold began to manifest themselves during autumn cold acclimation, with higher expression observed in the more resistant cultivar (Gaudet et al., 2000).

D.P. Livingston et al. (2021) found that cold acclimation of young wheat plants enhances the expression of genes coding for chitinase-1 (TaCHT-1) and IRIPs. However, the relationship between the freezing temperature of leaves from pre-acclimated plants and the expression levels of these genes turned out to be negative, i. e. the expression was lower in leaves that froze at lower temperatures.

Since plant AFPs lower the freezing point by only a fraction of a degree (Tremblay et al., 2005), the absence of a direct positive correlation between TaIRI-1 and TaCHT-1 expression and leaf freezing temperature is understandable. Expression levels of TaIRI-1 and TaCHT-1 show positive correlation with the abundance and complexity of bacterial and fungal communities, likely due to the structural and functional similarities between AFPs and PRPs.

These findings indicate the existence of differential expression of PRP genes regulating their activity depending on the wheat cultivar’s cold tolerance, the duration and direction of exposure, and organ-specific features.

G. Vaitkevičiūte et al. (2024) studied the effects of cold deacclimation and reacclimation on genome activity in frost-hardy and frost-sensitive T. aestivum cultivars. It was shown that a significant number of genes associated with pathogen response reacted to both deacclimation and repeated cold exposure, with gene activity affected by the duration of exposure. For example, both shortand long-term deacclimation of crowns led to reduced activity of the thaumatin-like protein gene, while longer exposure additionally reduced the activity of chitinaseencoding genes

During reacclimation, differential expression of genes coding for PRPs with similar action was observed. Among the genes coding for chitinase in crowns, some exhibited decreased activity during reacclimation, while others showed increased activity. Reacclimation in crowns also resulted in differential activity in thaumatinlike protein genes depending not only on the direction of change but also on the duration of exposure.

A significantly more diverse set of genes coding for thaumatin-like protein and chitinase in T. aestivum leaves compared to crowns responded with differential expression to deacclimation and reacclimation depending on the duration of exposure. The activity of PR genes in the crowns of the resistant cultivar Lakaja DS was less affected by temperature fluctuations compared to the more sensitive cultivar KWS Ferrum. In contrast, a greater number of genes were regulated by temperature fluctuations in the leaves of the more resistant cultivar compared to the more sensitive one, many of them being thaumatin-like protein genes (Vaitkevičiūte et al., 2024). These findings reveal a link between wheat cold tolerance and the transcription of PRP genes presumably also acting as IRIPs.

Given the above, we can conclude that wheat genes coding for PRPs are activated by cold and reacclimation and contribute to enhanced cold and frost hardiness. At the same time, genes belonging to the same PRP subgroup (chitinases, thaumatin-like proteins) are regulated differentially, and not all of them are activated by cooling

## Regulatory pathways of T. aestivum IRIP and cold-induced PRP gene expression

It has been shown that wheat IRI genes begin to express in response to decreasing temperatures. The activity of plant genes responsible for cold tolerance is regulated through both ABA-dependent and ABA-independent pathways. Among the latter, the ICE–CBF–COR signaling pathway is considered the primary one. This includes cold-regulated genes (COR – COld Related), which activated by C-repeat Binding Factor (CBF) with the activity of CBF controlled by a dedicated inducer (ICE – Inducer of CBF Expression) (Chinnusamy et al., 2003). The role of this pathway in activating cold adaptation genes in T. aestivum is widely recognized. However, the regulation of IRI genes by the ICE–CBF– COR transcription factor group has not been definitively proven, despite the fact that ICE proteins in T. aestivum bind to MYC elements (Badawi et al., 2008) present in TaIRI promoters (Jin et al., 2022). The literature search revealed one research suggesting a possible link between the ICE–CBF–COR pathway and IRI regulation in T. aestivum (Tchagang et al., 2017).

Another known regulatory pathway for cold adaptation is ABA-dependent (Xue-Xuan et al., 2010). Although ABA plays an important role in the development of cold and frost hardiness in T. aestivum (Zheng et al., 2020), some factors responsible for these traits are activated independently of ABA (Wang W. et al., 2017). The ABA involvement in the activation of TaIRI and PRP genes has not been confirmed, although TaIRI gene promoters contain elements associated with ABA-responsiveness (Jin et al., 2022). No activation of the TaIRI1 and TaIRI2 genes was observed after spraying 6-day-old T. aestivum seedlings with ABA (Tremblay et al., 2005).

In T. monococcum, ABA levels in leaves and crowns increase during the initial phase of non-lethal cold exposure, but the adaptation begins on the third day of exposure. Notably, the decrease in ABA levels coincides with an increase in salicylic acid content. Thus, it is possible that ABA’s influence on TaIRI and cold-induced PRP gene expression during cold exposure is limited based on exposure duration, and ABA may not be involved in the adaptive response during later phases requiring IRIP accumulation, when the regulatory role in adaptive changes may shift to other factors (Vanková et al., 2014).

Wheat IRIPs are structurally similar to PRPs, and its PRPs (β-1,3-glucanases, thaumatin-like proteins, and chitinases) also exhibit AFP properties. It is reasonable to assume that PR-associated factors, particularly the WRKY transcription factor family, may act as gene transcription factors for plant AFPs (Pandey, Somssich, 2009). Several findings indirectly support this possibility. For example, WRKY gene expression is associated with increased wheat resistance during the early stages of cold acclimation (Talanova et al., 2008; Winfield et al., 2010); some WRKY genes are activated by cold only in winter wheat (Winfield et al., 2010); and WRKY gene activity increases at subzero temperatures and depends on exposure duration (Herman et al., 2006). In addition, some wheat genes coding for glucanases, chitinases, and thaumatin-like proteins are co-regulated with genes coding for WRKY transcription factors (Winfield et al., 2010). The TaWRKY1 gene is activated by both pathogen attack and cold (Wang W. et al., 2017), and some WRKY proteins exhibit IRIP-like properties on their own (Huang, Duman, 2002).

It was shown that phytohormones involved in pathogen response, such as ethylene, jasmonic acid, and salicylic acid, also participate in the AFP gene regulation. These phytohormones are also involved in regulating IRI gene activity in wheat (Tremblay et al., 2005), although their role in cold and frost hardiness is not always definitively confirmed (Kurepin et al., 2013). In addition, it has been shown that ethylene is not associated with wheat cold tolerance (Macháčcková et al., 1989).

Expression of the TaIRI17, TaIRI18, and especially TaIRI16 genes in stem, root, and leaf tissues of young T. aestivum plants increased after exogenous treatment of wheat shoots, roots, and leaves with methyl jasmonate combined with cold exposure (+4 °C, 24 h) (Jin et al., 2022). Exogenous methyl jasmonate treatment also stimulated the activity of TaIRI gene promoters (Jin et al., 2022). However, jasmonic acid had different effects on TaIRI1 and TaIRI2 expression in 6-day-old seedlings of a frost-hardy T. aestivum cultivar, it induced TaIRI1 expression but not TaIRI2 (Tremblay et al., 2005). Analysis of motif activity of TaIRI gene promoters in tobacco and their expression in A. thaliana after the respective transformations showed that MYB, Mybb, MYC, CGTCA, and TGACG motifs from the TaIRIp1 promoter were highly active and inducible by cold and methyl jasmonate (Jin et al., 2022).

The role of salicylic acid in AFP gene regulation still remains to be confirmed. No activation of TaIRI1 and TaIRI2 was observed after spraying 6-day-old seedlings of the frost-hardy T. aestivum cultivar with salicylic acid (Tremblay et al., 2005). Treatment of young wheat plants of cultivar Yangmai 16 with salicylic acid solution followed by 6 days of field freezing (at the average temperatures of +5.3 °C and minimum of –7 °C) did not increase IRI2 (AY968589) expression compared to control temperature conditions (average +12 °C) (Wang W. et al., 2021). At the same time, freezing without salicylic acid solution treatment led to a twofold increase in gene expression compared to control. According to the authors, previous experiments showed that pretreatment with salicylic acid could increase TaIRI2 expression during 48-hour cold exposure (Wang W. et al., 2020). Thus, it may be suggested that salicylic acid regulation of TaIRI2 expression may depend on exposure duration, though this requires further study

Ethylene is involved in regulating antifreeze activity in winter rye in response to cold and drought, with β-1,3-glucanases, chitinases, and thaumatin-like proteins accumulating in the leaf apoplast (Yu et al., 2001). In T. aestivum, spraying 6-day-old seedlings of a frosthardy cultivar with ethylene solution led to differential regulation of the TaIRI1 and TaIRI2 genes: specifically, it induced TaIRI1 expression but not that of TaIRI2 (Tremblay et al., 2005). However, ethylene turned out to have a negative association with wheat cold tolerance (Macháčcková et al., 1989).

Overall, data on transcription factor involvement in AFP gene expression regulation remain limited, and information on phytohormonal regulation is often contradictory. It appears that the duration, intensity, and phase of cold exposure play a significant role in phytohormonal regulation. It also remains unclear why the studied phytohormones do not enhance wheat cold tolerance despite their ability to activate TaIRI genes. This aspect of antifreeze activity regulation requires further investigation.

## Practical application of T. aestivum IRIPs and cold-induced PRPs

The use of plant AFPs is limited by their reduced thermal hysteresis capacity compared to animal AFPs and is primarily justified by their ice recrystallization inhibition (IRI) activity. One of the most promising directions for the practical application of AFPs is the development of more frost-hardy plants and cultivars. Although gene transfer studies involving wheat AFPs have yielded encouraging results (Jin et al., 2022), practical use of the available knowledge on wheat IRIPs remains limited.

In present, the main area of practical application for plant AFPs is cryopreservation in medicine and food industry. Naturally derived AFPs are less toxic than dimethyl sulfoxide (DMSO), which is commonly used today, making them promising cryoprotectants (Chow-Shi-Yée et al., 2020). A crude extract from wheat seedlings turned out to protect rat hepatocytes during cryopreservation; the effect could be successfully enhanced by further purification of the extract or by using recombinant TaIRI-2 protein, which enabled longer storage of hepatocytes and recovery of a greater number of viable cells restoring their functions upon thawing (Grondin et al., 2009).

When recombinant TaIRI-2 was used for cryopreserving insulin-secreting INS832/13 cells, it was found that TaIRI-2 could penetrate these cells as well as hepatocytes. This likely contributed to improved cell survival after thawing and reduced cell death (Chow-Shi-Yée et al., 2016). However, it is important to consider that plant-based cryoprotectants may have immunogenic properties, potentially triggering unwanted immune or allergic reactions, which is a significant limitation for medical cryopreservation uses (Kostyaev et al., 2016)

In the food industry, natural AFPs are widely used in ice cream production, with several patents granted, including one for the use of AFPs from winter rye (Boonsupthip, Lee, 2003). AFPs extracted from coldacclimated wheat significantly improved ice cream texture, and this effect was not diminished by shock freezing (Regand, Goff, 2006). Adding AFPs extracted from flour of frost-hardy winter wheats to bread dough reduces water flow and migration during freeze-thaw cycles, helping to preserve the dough’s internal structure and gluten network, increase viscosity and elasticity, and improve textural properties (Wang X. et al., 2024).

Direct use of raw plant extracts in the food industry is preferable to using purified extracts due to their low yield and the complexity of the purification process. However, using concentrated extracts can be problematic due to the presence of enzymes that may negatively affect the taste or texture of the final product. Undesired enzymatic activity in the extract can be eliminated by heat treatment, so thermal stability becomes necessary for natural AFPs used in food production. A promising thermostable thaumatin-like AFP was identified in the leaf apoplast extract of cold-acclimated T. aestivum (Kontogiorgos et al., 2007).

## Conclusion

The relationship between the expression of AFP genes (with the subsequent accumulation of their respective products) and increased frost hardiness in winter wheat has been convincingly demonstrated in a number of model experiments. It has been shown that the accumulation of apoplastic proteins and AFPs correlates with the survival of winter wheat plants under field conditions (Chun et al., 1998). However, it remains unclear how significant the contribution of antifreeze activity is to plant survival compared to other known factors (e. g., accumulation of free proline and oligosaccharides, depth of occurrence of the crown). Therefore, including AFP accumulation as a target trait in breeding programs still requires more solid justification. Wheat AFPs exhibit dual antifreeze and antipathogenic activity. On the one hand, this duality complicates the assessment of the specific contribution of AFPs to wheat survival; on the other hand, it makes the presence of this trait particularly valuable, as it reflects the presence of two types of resistance and yield-enhancing factors.

In the vast majority of model experiments, the effect of low positive temperatures on the expression of IRIP and cold-induced PRP genes was assessed, while only a few studies examined the impact of subzero temperatures (Herman et al., 2006; Kang et al., 2013; Willick et al., 2018). As a result, researchers can currently draw conclusions only about the association of these genes with cold tolerance. However, the actual need for these proteins arises under freezing conditions. Simulating field-like subzero temperatures in experimental setups could not only deepen our understanding of IRIP and PRP gene expression but also bring us closer to understanding how their functions are realized in real-world conditions.

Understanding the regulatory mechanisms controlling AFP gene activity remains a relevant problem, as the overall picture of their regulation is still unclear, especially regarding the protein factors involved in gene expression. The hormonal regulation patterns of wheat AFPs are also not fully understood. It is highly likely that the dual nature of plant AFPs has led to the development of a regulatory system in which signaling pathways for both biotic and abiotic stress responses are involved. This hypothesis requires particularly thorough investigation.

The promising results of practical applications of wheat AFPs, along with evidence linking cold tolerance, gene expression, and protein accumulation in winter wheat, support the need for continued research into this group of low-temperature adaptation proteins

## Conflict of interest

The authors declare no conflict of interest.
